# Microstructural, Mechanical, and Tribological Performances of Composites Prepared via Melt Compounding of Polyamide 6, Basalt Fibers, and Styrene–Ethylene–Butylene–Styrene Copolymer

**DOI:** 10.3390/ma16083237

**Published:** 2023-04-19

**Authors:** Qiaolie Zheng, Bin Wang, Xiping Li, Xiangde Xiao, Huimei Jin, Hongwei Zhang, Yuan Zhao

**Affiliations:** 1 Key Laboratory of Urban Rail Transit Intelligent Operation and Maintenance Technology & Equipment of Zhejiang Province, College of Engineering, Zhejiang Normal University, Jinhua 321004, China; 2 Jinhua Hefa Technology Co., Ltd., Jinhua 321004, China

**Keywords:** polymer composites, mechanical property, friction, rheological behavior

## Abstract

Basalt fibers (BFs) are environmentally friendly materials characterized by high strength and good wear resistance, and thus are popular candidates for reinforcing polymers. Herein, polyamide 6 (PA 6), BFs, and the styrene–ethylene–butylene–styrene (SEBS) copolymer were melt compounded sequentially to prepare fiber-reinforced PA 6-based composites. The results showed improved mechanical and tribological performances via the incorporation of BFs and SEBS into PA 6. Compared to neat PA 6, an average 83% increase in notched impact strength was achieved for the PA 6/SEBS/BF composites, which is mainly due to the good miscibility between SEBS and PA 6. The tensile strength of the composites, however, was only increased moderately, since the weak interfacial adhesion was not sufficiently efficient to transfer the load from the PA 6 matrix to the BFs. Interestingly, the wear rates of both the PA 6/SEBS blend and the PA 6/SEBS/BF composites were obviously lower than those of the neat PA 6. The PA 6/SEBS/BF composite with 10 wt.% of the BFs exhibited the lowest wear rate of 2.7 × 10^−5^ mm^3^/N·m, which was decreased by 95% compared to that of the neat PA 6. The facilitation of forming tribo-film with SEBS and the naturally good wear resistance of the BFs were responsible for the largely decreased wear rate. Moreover, the incorporation of SEBS and BFs into the PA 6 matrix transformed the wear mechanism from adhesive wear to abrasive wear.

## 1. Introduction

Fiber-reinforced polymer composites (FRPs) are a continuous topic in both the academy and industry [1,2,3]. Among the various FRPs, fiber-reinforced polyamide 6 (PA 6) composites attract the most attention in the field of engineering, due to their advantages of high stiffness, high toughness and light weight [4,5]. For example, glass fiber-reinforced PA 6 composites exhibit significantly enhanced mechanical properties, dimensional stability, and wear resistance compared to neat PA 6 [6,7]. Thus, glass fiber-reinforced PA 6 composites can partially replace metals and ceramics for some engineering applications [8].

Recently, a kind of environmental friendly natural fiber, basalt fiber (BF), has attracted increasing attention as a reinforcing material. As reported in the literature, BF is composed of 43–53% SiO_2_, 12–16% Al_2_O_3_, 6–18% iron oxide, 10–20% alkaline earth metal, and 2–8% alkali [9]. Compared to glass fibers, BF exhibits better mechanical properties, soundproof performance, and heat, abrasion and chemical resistance [10]. Thus, BFs are anticipated to replace glass fibers for reinforcing polyamides. Yu et al. prepared reinforced PA 66 composites using silane-functionalized BFs [11,12]. The composites were injection molded into the mold to form standard specimens for mechanical property testing. It was found that significantly enhanced tensile strength and flexural strength were achieved via incorporating BFs.

Apart from the mechanical strength, the wear resistance of the polyamide material is another crucial factor for its application in the engineering field [13]. It is reported that the tribological performance of the polyamide material could be significantly improved via incorporating glass fibers, carbon fibers, or BFs [14,15,16]. The intrinsic high modulus and high wear resistance of the reinforcing fibers as well as the good interfacial adhesion were ascribed to the enhanced tribological performance [17]. Additionally, the relatively soft materials may tend to form a protective tribofilm under friction, which also helps improve tribological performance [18]. This naturally leads to the idea that tribological performance would be highly enhanced via simultaneously incorporating BFs and a soft elastomer into the PA 6 matrix.

As to the manufacture of fiber-reinforced polymer composites, 3D printing provides much more design freedom than traditional molding technologies do [19,20]. It can be expected that various BF-reinforced PA 6 composite engineering parts will be manufactured via 3D printing in the next few years. This inspires the authors to explore the adaptability of BF-reinforced PA 6 composites for 3D printing. However, it is well known that polymer composites with a high content (≥15 wt.%) of fibers are quite difficult to be processed into filaments for 3D printing. Thus, in this work, the authors introduce a self-developed extrusion-based 3D printer, and it is tested for the manufacture of the BF-reinforced PA 6 composite specimens. Both mechanisms of mechanical and tribological performance improvement are explored and revealed.

## 2. Experimental Part

### 2.1. Materials

PA 6 granules (grade J-2400) were purchased from Hangzhou Juheshun New Material Co., Ltd., Hangzhou, China. The number-average molar mass of the PA 6 granules was 14,500 g/mol. Maleic anhydride-grafted styrene–ethylene–butylene–styrene copolymer (SEBS) granules with a melt flow rate of 5.0 g/10 min (230 °C, 2.16 kg) were purchased from Guangzhou Yuehuacheng New Material Co., Ltd., Guangzhou, China. The molar content of maleic anhydride in the SEBS granules was 1.8%. Chopped basalt fibers were kindly supplied by Shijin Basalt Fiber New Materials Co., Ltd., Hangzhou, China.

### 2.2. Melt Compounding and Sample Preparation

**Figure 1:**
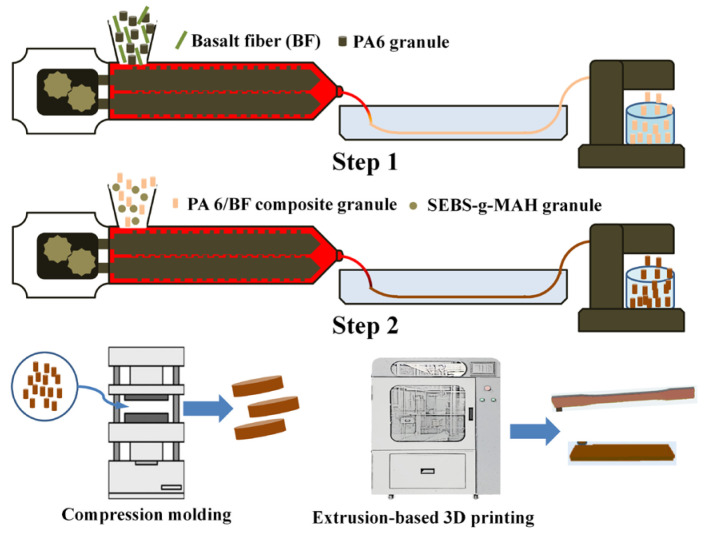
Schematics of melt compounding technique and sample preparation of PA 6/SEBS/BF composites.

Herein, a two-step method was applied to prepare the PA6/SEBS/BF composites, as is shown Figure 1.

Firstly, the PA 6 granules and BF were compounded using a twin screw extruder (HAAKE Process 11) at a screw rotation speed of 80 r/min. The temperatures were set as 220 °C, 230 °C, 235 °C, 235 °C, 235 °C, 235 °C, and 230 °C from hopper to die. Before melt compounding, the PA 6 granules were dried in a vacuum oven for 12 h at 90 °C. Secondly, the extruded PA 6/BF composites were compounded with SEBS granules using the twin screw extruder. The weight ratio of PA 6 and SEBS was set at 85/15. Then, PA 6/SEBS/BF composites with three different contents of BF (5, 10, and 20 wt.%) were obtained via controlling the weight ratio of PA 6 and BF during the first extrusion. For comparison, the neat PA 6 and PA 6/SEBS blend were also prepared using the two-step method. The prepared PA 6/SEBS/BF composites were denoted as PA 6/SEBS/BFx, where x indicates the weight fraction of BF.

After being vacuum-dried at 90 °C for 12 h, the PA 6/SEBS/BF composites, PA 6/SEBS blend, and neat PA 6 were compression molded into disc samples of a diameter of 25 mm and a thickness of 2 mm, at 230 °C and 10 MPa. The standard samples for tensile and impact tests were 3D printed using an extrusion-based 3D printer (CI Uni-Print 450, Zhejiang Challenge & Intelligence Technology, Co., Ltd., Jinhua, China).

### 2.3. Characterization

Rheological measurements were carried out at 225 °C under nitrogen flow, using a rotational rheometer (MCR702, Anton Paar Instrument, Graz, Austria). By using parallel-plate geometry (a diameter of 25 mm and a gap of 1 mm), the dynamic oscillatory shear mode was selected for the tests. The frequency range was set between 0.01 and 100 Hz, and the strain was 0.5%.

Rectangle specimens were cut from the PA 6/SEBS/BF composite disc samples. Then, the specimens were cryo-fractured in liquid nitrogen and were gold sputtered, followed by examination via a scanning electron microscopy (SEM) instrument (EM-30PLUS, COXEM, Silicon Daejeon, South Korea.). The worn surfaces of the PA 6/SEBS/BF composites after the friction tests were also examined using the SEM instrument.

Tensile tests were carried out using a universal testing machine (UTM 4024, SUNS, Shenzhen, China.) at a strain rate of 5 mm/min at room temperature. Each experiment was repeated five times. A Vicker’s square-based diamond indenter (TMVS-S1-ALC, Beijing Shidai Sihe Technology, Co., Ltd., Bejing, China.) was employed to measure the microhardness from the residual impression on the sample surface after an indentation time of 10 s. Loads of 0.0980 N were used to derive the value of microhardness in MPa by the following equation [21]:

(1) $$H=K\frac{P}{{}^{d^{2}}}$$

where *d* (m) is the length of the indentation diagonal, *P* (N) is the applied load, and K is a geometrical factor (1.854). At least ten imprints were taken on the surface of the disc samples for each load.

The tribological and wear performances of the PA 6/SEBS/BF composites were determined by a tribometer (MFT-5000, Rtec, San Jose, CA, USA) with a ball-on-disk configuration. A steel ball (GCr15, 61 HRC) of a diameter of 10 mm and with a load of 20 N was used as a static counter body. The tribological tests were conducted at an oscillation frequency of 6 Hz and sliding amplitude of 4 mm. A white-light interferometer (Contour GT-X, Bruker, Billerica, MA, USA.) was employed to analyze the wear volume of the wear tracks after the friction tests. The specific wear rate was calculated according to the following equation:

(2) $$R=\frac{\Delta V}{PL}$$

where Δ*V*, *P*, and *L* represent the wear volume loss, pressure, and sliding distance, respectively. Prior to the test, the pin was polished with waterproof abrasive papers to obtain a Ra of ≤0.1 μm.

## 3. Results and Discussion

### 3.1. Linear Rheological Behavior of PA 6/SEBS/BF Composites

**Figure 2:**
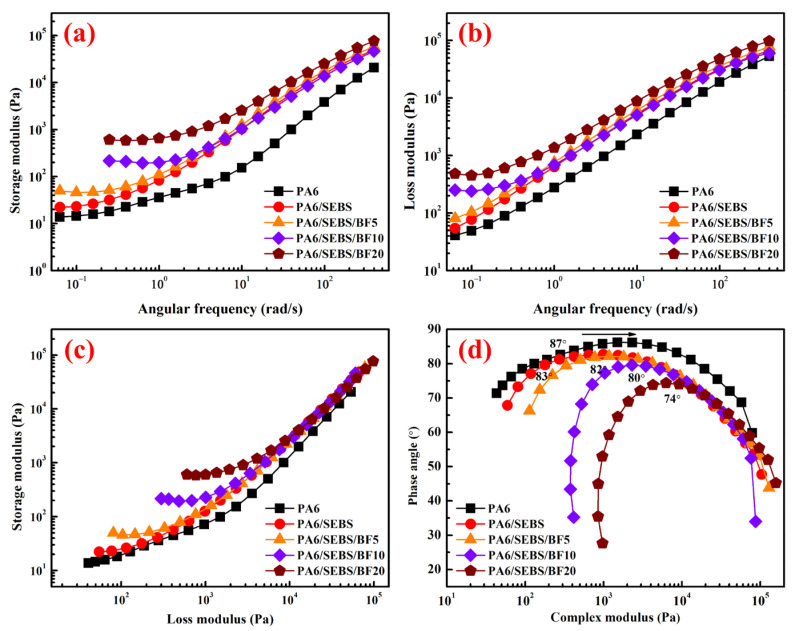
(**a**) Storage modulus versus frequency curves, (**b**) loss modulus versus frequency curves, (**c**) Han plots, and (**d**) van Gurp−Palmen plots of PA 6/SEBS/BF composites.

Figure 2a,b shows the storage modulus vs. the frequency and loss modulus vs. the frequency curves of the PA 6/SEBS/BF composites.

As can be seen in Figure 2a, the storage modulus at both low and high frequencies of PA 6 is increased with the incorporation of SEBS. As reported by Sailer and Handge, the enhancement in the storage modulus can be ascribed to the strengthened interfacial adhesion, which results from the reaction between the amino end group of PA 6 and the maleic anhydride of SEBS [22]. The storage modulus is observed to further increase with the incorporation of BF. A plateau appears in the low-frequency range of the PA 6/SEBS/BF20 composite. As has been reported in the literature [23], the appearance of a plateau reflects the formation of filler networks in the composites.

To further analyze rheological behavior, the Han and van Gurp−Palmen (vGP) plots were introduced, which are shown in Figure 2c,d. The Han plots were reported to be composition-independent for compatible systems while they turned dependent for immiscible ones [24]. As can be deduced from the Han plots in Figure 2c, the PA 6 and SEBS exhibit good compatibility due to the effect of interfacial reactive compatibilization. However, the incorporation of BF led to large-scale heterogeneity in the composites, as evidenced by the obvious reduction in the slope in the terminal region. For the vGP plots, as can be seen in Figure 2d, the plateau of the phase angle exhibits an obvious decrease (to 74°) for the PA 6/SEBS/BF20 composite, reflecting the formation of BF networks [25].

### 3.2. Microstructure of PA 6/SEBS/BF Composites

**Figure 3:**
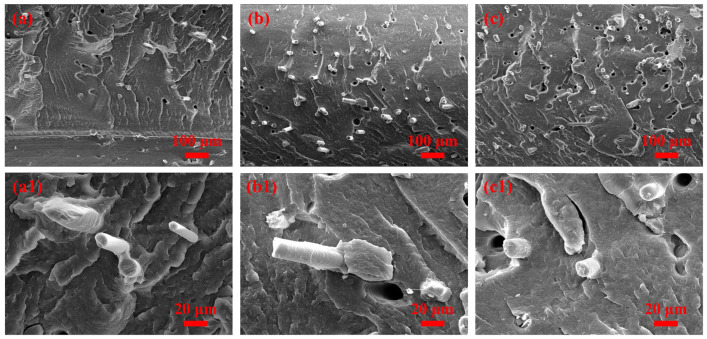
SEM micrographs of cryo-fractured surfaces of PA 6/SEBS/BF composites with BF contents of (**a**) 5, (**b**) 10, and (**c**) 20 wt.%; (**a1**), (**b1**), and (**c1**) are magnified SEM micrographs from (**a**), (**b**), and (**c**), respectively.

Figure 3 shows the SEM micrographs of the cryo-fractured surfaces of the PA 6/SEBS/BF composites.

Both BFs and holes are observed in the PA 6 matrix. The holes were formed due to the fact that BFs were pulled out from the PA 6 matrix. One can observe from Figure 3a–c that the BFs were uniformly dispersed in the PA 6/SEBS/BF composites, regardless of the BF content. This can be ascribed to the low tendency of aggregation of bare BFs and shear effect during melt compounding [26,27].

The magnified SEM micrographs for the three PA 6/SEBS/BF composites are shown in Figure 3a1–c1, respectively. One can observe that both the surfaces of the BFs and hole were smooth, indicating relatively weak interfacial adhesion between the BFs and the PA 6 matrix. One may note that the SEBS droplets are not observed even in the magnified SEM micrographs. This can be ascribed to the good miscibility between the SEBS and PA 6, so the dispersed SEBS droplets are too small to be easily distinguished with the magnification used in this work.

### 3.3. Mechanical Performance of PA 6/SEBS/BF Composites

**Figure 4:**
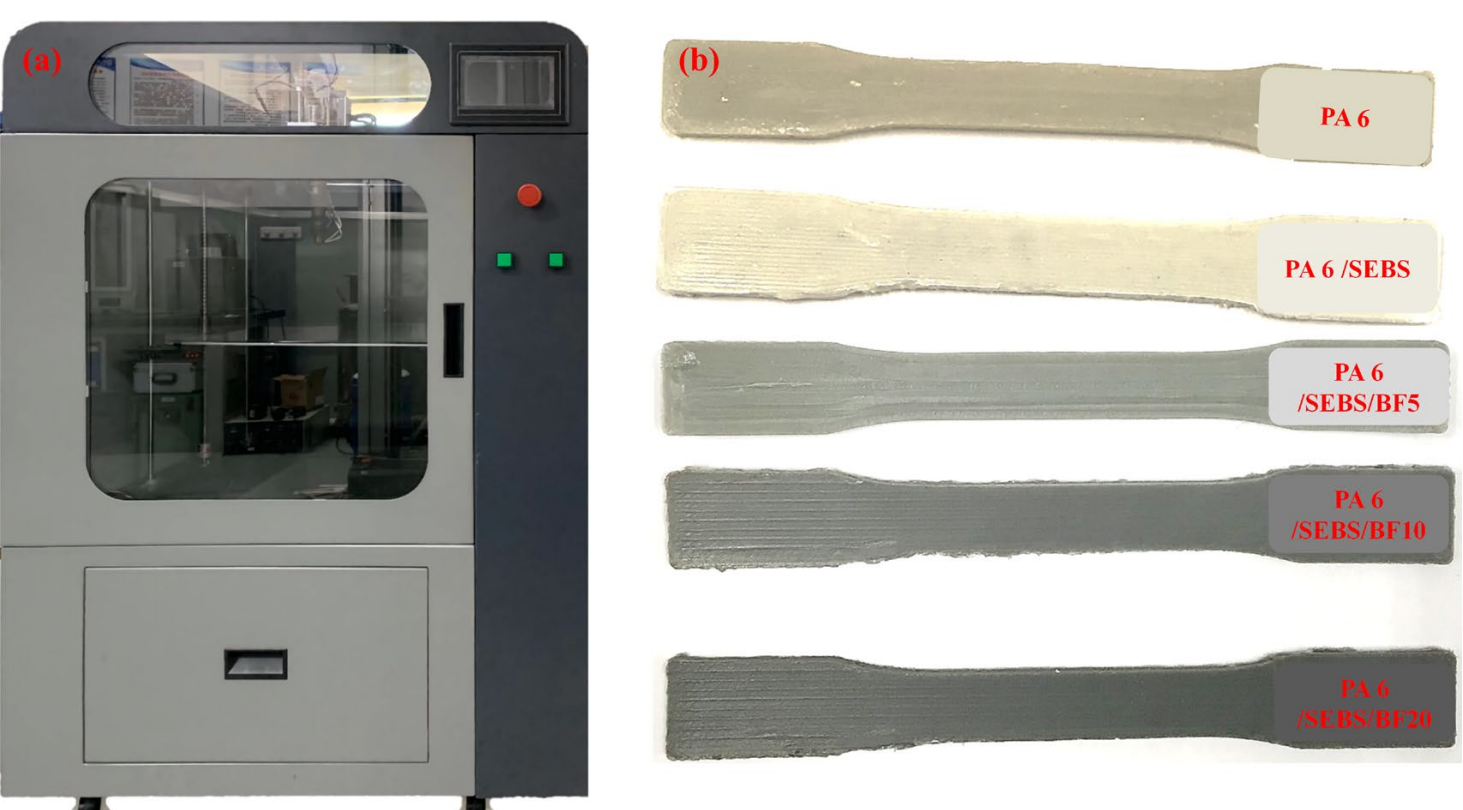
Electronic images of (**a**) self-developed extrusion-based 3D printer and (**b**) 3D-printed tensile specimens.

The electronic images of the extrusion-based 3D printer and the printed standard tensile specimens are shown in Figure 4.

The extrusion-based 3D printer was self-developed by our group and commercialized by Zhejiang Challenge & Intelligence Technology, Co., Ltd. It has a single-screw extruder system with a custom-designed 18 mm diameter screw. Detailed information for the 3D printer is reported in our previously published work [28]. As can be seen in Figure 4b, from the neat PA 6 to the PA 6/SEBS/BF20 composite, standard tensile specimens are all successfully printed at a high quality using our extrusion-based 3D printer. This may be direct evidence that the extrusion-based 3D printer is more materials available than the filament-based 3D printer does.

**Figure 5:**
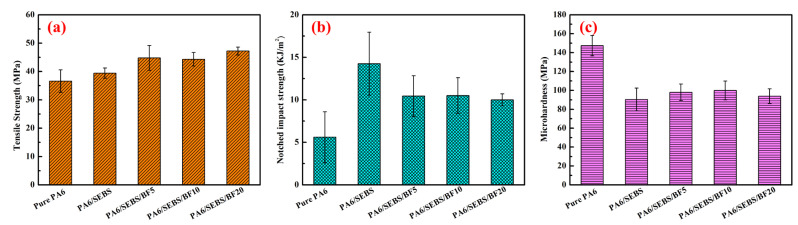
(**a**) Tensile strength, (**b**) notched impact strength, and (**c**) microhardness of neat PA 6, PA 6/SEBS blend, and PA 6/SEBS/BF composites.

The tensile strength, notched impact strength, and microhardness of the PA 6/SEBS/BF composites are exhibited in Figure 5.

It can be observed from Figure 5a that the neat PA 6 exhibits an average tensile strength of 36.6 MPa, which is far lower than that of the injection molded one [29]. The lower tensile strength can be due to the less compacted structure in 3D-printed products [30]. One may note that the average tensile strength of the PA 6/SEBS blend is higher than that of the neat PA 6. However, the tensile strength of semi-crystalline polymers is usually lowered by the incorporation of elastomers [31]. Herein, for the situation of 3D printing, the incorporation of SEBS may have helped improve the bonding of printed layers. Thus, the tensile strength was enhanced. The tensile strength is observed to further increase with the incorporation of BFs. The average tensile strength of the PA 6/SEBS/BF composites is around 45 MPa, regardless of the BF content. The average improvement of 23% in tensile strength is attributed to the reinforcing effect of BFs [32]. However, the reinforcing effect of bare BFs is relatively weak, due to the weak interfacial adhesion.

It is well known that the impact strength of semi-crystalline polymers can be improved via blending with certain elastomers [33,34]. In this work, the notched impact strength of neat PA 6 is observed to significantly improve from 5.4 to 14.3 KJ/m^2^ with the incorporation of SEBS, as is shown in Figure 5b. This can be ascribed to the elastomeric nature of SBES and its uniform dispersion in the PA 6 matrix, as evidenced by the Han curves (Figure 2c). Further incorporation of BFs led to a slight decrease in the notched impact strength, due to the weak interfacial adhesion between BFs and the PA 6 matrix. Moreover, one can note that the BFs experienced twice the extrusion for preparing the PA 6/SEBS/BF composites. The superimposed intensive shear effects are expected to drastically decrease the average length of BFs. As a result, the reinforcing effects of BFs on both tensile and impact strength would be partially sacrificed.

Microhardness is an important indicator of the surface mechanical performance of materials [35]. As is shown in Figure 5c, neat PA 6 exhibits an average microhardness of 147.3 MPa. The average microhardness sharply decreased to 90.5 MPa with the incorporation of SEBS, due to the very soft nature of SEBS and its uniform dispersion in the PA 6 matrix. The average microhardness moderately increased to 98.6, 100, and 94 MPa with the addition of 5, 10, and 20 wt.% BFs, respectively. The much lower average microhardness of the PA 6/SEBS/BF composites than that of neat PA 6 is also a sign of the uniform dispersion of SEBS.

### 3.4. Tribological Performance of PA 6/SEBS/BF Composites

**Figure 6:**
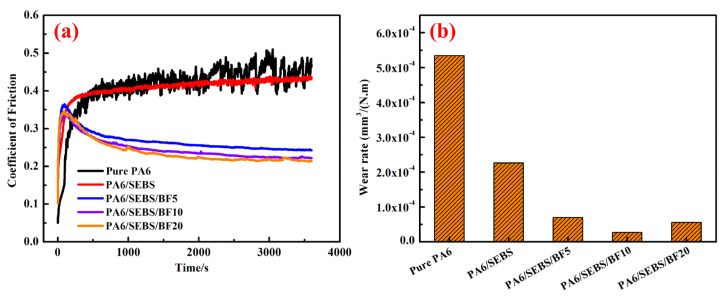
(**a**) The coefficient of friction and (**b**) wear rate of neat PA 6, PA 6/SEBS blend, and PA 6/SEBS/BF composites.

Figure 6a shows the friction coefficient of PA 6/SEBS/BF composites as a function of time.

It is observed that both the neat PA 6 and PA 6/SEBS blend exhibit nearly stable friction coefficient curves. The neat PA 6 shows a coefficient of friction of about 0.43, corresponding with the reported one in the literature [36]. For the PA 6/SEBS blend, its coefficient of friction is similar to that of the neat PA 6. The friction coefficient curves of the PA 6/SEBS/BF composites, however, exhibit a sharp decrease region followed by a long nearly stable region. In the nearly stable region, the coefficient of frictions for the PA 6/SEBS/BF composites with the addition of 5, 10, and 20 wt.% BFs are about 0.25, 0.23, and 0.22, respectively. The significant decrease in the coefficient of friction can be attributed to the incorporation of BFs.

**Figure 7:**
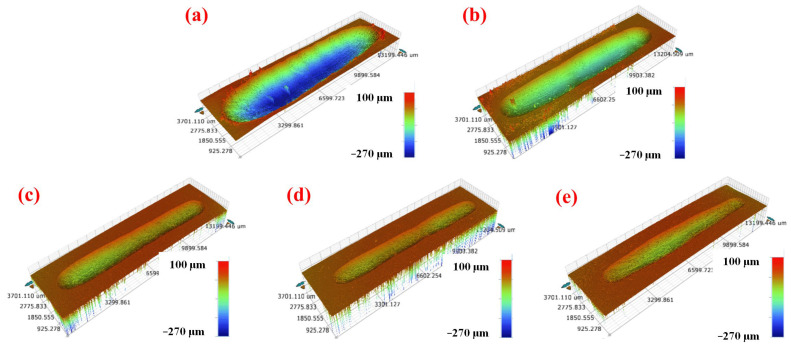
Three-dimensional morphologies of the worn surface of (**a**) neat PA 6, (**b**) PA 6/SEBS blend, (**c**) PA 6/SEBS/BF5, (**d**) PA 6/SEBS/BF10, and (**e**) PA 6/SEBS/BF20.

The wear rate of the PA 6/SEBS/BF composites is shown in Figure 6b. It is noted that PA 6/SEBS/BF composites exhibit a much lower wear rate than the neat PA 6 does. The wear rate of the neat PA 6 and PA 6/SEBS blend is 5.3 × 10^−4^ and 2.3 × 10^−4^ mm^3^/N·m, respectively. These values are largely decreased to 7.0 × 10^−5^, 2.7 × 10^−5^, and 5.6 × 10^−5^ mm^3^/N·m for PA 6/SEBS/BF5, PA 6/SEBS/BF10, and PA 6/SEBS/BF20, respectively. The obvious difference in wear rates can be clearly reflected by their wear scars, as are shown in Figure 7.

The neat PA 6 shows a wide and rough wear scar, with the largest depth of 270 μm (Figure 7a). In the presence of SEBS, the wear scar becomes shallow and narrow (Figure 7b), indicating the effect of a lower wear rate by the addition of SEBS. For the PA 6/SEBS/BF composites, the wear scars are much shallower and narrower than the wear scar of the neat PA 6. Among the composites, PA 6/SEBS/BF10 exhibits the shallowest and narrowest wear scar, which corresponds with its lowest wear rate, which is shown in Figure 6b.

**Figure 8:**
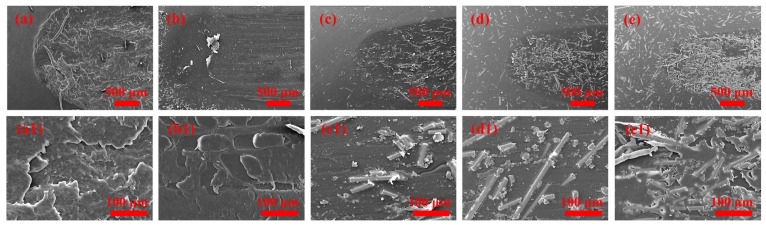
SEM micrographs of (**a**) neat PA 6, (**b**) PA 6/SEBS blend, (**c**) PA 6/SEBS/BF5, (**d**) PA 6/SEBS/BF10, and (**e**) PA 6/SEBS/BF20; (**a1**–**e1**) corresponding magnified SEM micrographs for neat PA 6 to PA 6/SEBS/BF20.

Figure 8 shows SEM micrographs of the worn surfaces of PA 6/SEBS/BF composites.

Obvious plastic deformation and the viscoelastic flow phenomenon are observed for the neat PA 6, suggesting the mainly adhesive wear mechanism [37]. Discontinuous grooves and wear debris are observed in the worn surface of the PA 6/SEBS blend, suggesting that the relatively soft SEBS formed discontinuous tribo-films during the friction test. For the PA 6/SEBS/BF, microcracks between the BFs and PA 6 matrix as well as grooves are observed in the worn surface, indicating abrasive and fatigue wear being the dominant wear mechanism. Comparing Figure 8d1,e1, one finds that BF clusters form in the worn surface of PA 6/SEBS/BF20, reflecting the easy formation of large wear debris. This is the reason that PA 6/SEBS/BF20 exhibits a lower wear resistance than PA 6/SEBS/BF10 does.

## 4. Conclusions

In summary, the PA 6/SEBS/BF ternary composites were prepared using a two-step melt compounding method. The bare BFs were uniformly dispersed in the PA 6 matrix, due to their low tendency of aggregation and the experienced two stages of shear. The SEBS exhibited good miscibility with the PA 6, thanks to the reaction between the maleic anhydride of SEBS and the amino end group of PA 6. As a result, the notched impact strength of the composites was significantly improved. However, only a moderate improvement was observed for the tensile strength, due to the weak interfacial adhesion between BFs and PA 6 matrix. The wear resistance of PA 6 was obviously improved with the incorporation of both SEBS and BFs, which transformed the wear mechanism from an adhesive to an abrasive one.

## Data Availability

Not applicable.
